# Differentiating a pachychoroid and healthy choroid using an unsupervised machine learning approach

**DOI:** 10.1038/s41598-022-20749-9

**Published:** 2022-09-29

**Authors:** Reza Mirshahi, Masood Naseripour, Ahmad Shojaei, Mohsen Heirani, Sayyed Amirpooya Alemzadeh, Farzan Moodi, Pasha Anvari, Khalil Ghasemi Falavarjani

**Affiliations:** 1 Eye Research Center, The Five Senses Institute, Rassoul Akram Hospital, Iran University of Medical Sciences, Sattarkhan-Niaiesh St., Tehran, 11335 Iran; 2 Stem Cell and Regenerative Medicine Research Center, Iran University of Medical Sciences, Tehran, Iran; 3 Basir Eye Health Research Center, Tehran, Iran; 4 Translational Ophthalmology Research Center, Farabi Eye Hospital, Tehran University of Medical Sciences, Tehran, Iran

**Keywords:** Retinal diseases, Machine learning

## Abstract

The purpose of this study was to introduce a new machine learning approach for differentiation of a pachychoroid from a healthy choroid based on enhanced depth-optical coherence tomography (EDI-OCT) imaging. This study included EDI-OCT images of 103 eyes from 82 patients with central serous chorioretinopathy or pachychoroid pigment epitheliopathy, and 103 eyes from 103 age- and sex-matched healthy subjects. Choroidal features including choroidal thickness (CT), choroidal area (CA), Haller layer thickness (HT), Sattler-choriocapillaris thickness (SCT), and the choroidal vascular index (CVI) were extracted. The Haller ratio (HR) was obtained by dividing HT by CT. Multivariate TwoStep cluster analysis was performed with a preset number of two clusters based on a combination of different choroidal features. Clinical criteria were developed based on the results of the cluster analysis, and two independent skilled retina specialists graded a separate testing dataset based on the new clinical criteria. TwoStep cluster analysis achieved a sensitivity of 1.000 (95-CI: 0.938–1.000) and a specificity of 0.986 (95-CI: 0.919–1.000) in the differentiation of pachy- and healthy choroid. The best result for identification of pachychoroid was obtained for a combination of CT, HR, and CVI, with a correct classification rate of 0.993 (95-CI: 0.980–1.000). Based on the relative variable importance (RVI), the cluster analysis prioritized the choroidal features as follows: HR (RVI: 1.0), CVI (RVI: 0.87), CT (RVI: 0.70), CA (RVI: 0.59), and SCT (RVI: 0.27). After performing a receiver operating characteristic curve analysis on the cluster membership variable, a cutoff point of 389 µm and 0.79 was determined for CT and HR, respectively. Based on these clinical criteria, a sensitivity of 0.793 (95-CI: 0.611–0.904) and a specificity of 0.786 (95-CI: 0.600–0.900) and 0.821 (95-CI: 0.638–0.924) were achieved for each grader. Cohen's kappa of inter-rater reliability was 0.895. Based on an unsupervised machine learning approach, a combination of the Haller ratio and choroidal thickness is the most valuable factor in the differentiation of pachy- and healthy choroids in a clinical setting.

## Introduction

Pachychoroid disease consists of a spectrum of retinochoroidal abnormalities, including central serous chorioretinopathy (CSC), pachychoroid pigment epitheliopathy (PPE), pachychoroid neovasculopathy, polypoidal choroidal vasculopathy (PCV)/aneurysmal type 1 neovascularization, focal choroidal excavation, and peripapillary pachychoroid syndrome^1^. This clinical spectrum shares common morphological changes in the choroidal structure, including choroidal thickening, choriocapillaris attenuation, and dilation of choroidal vessels. Despite regular encounters by retinal specialists with this entity, the literature lacks a uniform definition of pachychoroid. Although increased choroidal thickness and choroidal vascular dilation have been widely accepted as major diagnostic criteria for pachychoroid disease, the cut-off for choroidal thickness and the definition of choroidal pachyvessels is not clear^2^.

The sclero-choroidal junction can be easily visualized in enhanced-depth imaging optical coherence tomography (EDI-OCT), facilitating the measurement of choroidal thickness as a major index in the diagnosis of pachychoroid disease. In addition, different choroidal layers (Haller, Sattler, and choriocapillaris) are discernible with this imaging technique, and various imaging indices have been previously proposed for quantification of vascular dilation in EDI-OCT^3,4^.

With the advances in imaging techniques and the availability of complex data for different disease entities, data-driven approaches are gaining popularity in biomedical research. Unsupervised machine learning techniques help to differentiate features of similar diseases and to revise the diagnostic criteria. Cluster analysis is a method of unsupervised machine learning that classifies cases into different clusters without the need for prior labeling of the data groups. The members of each cluster are like each other but dissimilar to the members of other clusters. This method utilizes computer power and impartiality to find patterns previously missed in routine statistical analyses^5^. The hidden associations between patients with complex set of characteristics can be revealed by this analysis^6^. In addition it has been proved to be useful in developing clinical criteria in diseases with complex nature^7^ and to find differentiating features between two clinical entities^8^. This approach is new in ophthalmology, and limited studies reported the use of cluster analysis for differentiating features of ophthalmic diseases such as Fuchs’ uveitis syndrome, multifocal choroiditis and punctate inner choroidopathy.

The current study aims to use cluster analysis to find differentiating choroidal features between patients with CSC or PPE and healthy subjects and to develop clinical criteria based on EDI-OCT for the diagnosis of pachychoroid disease.

## Methods

**Figure 1:**
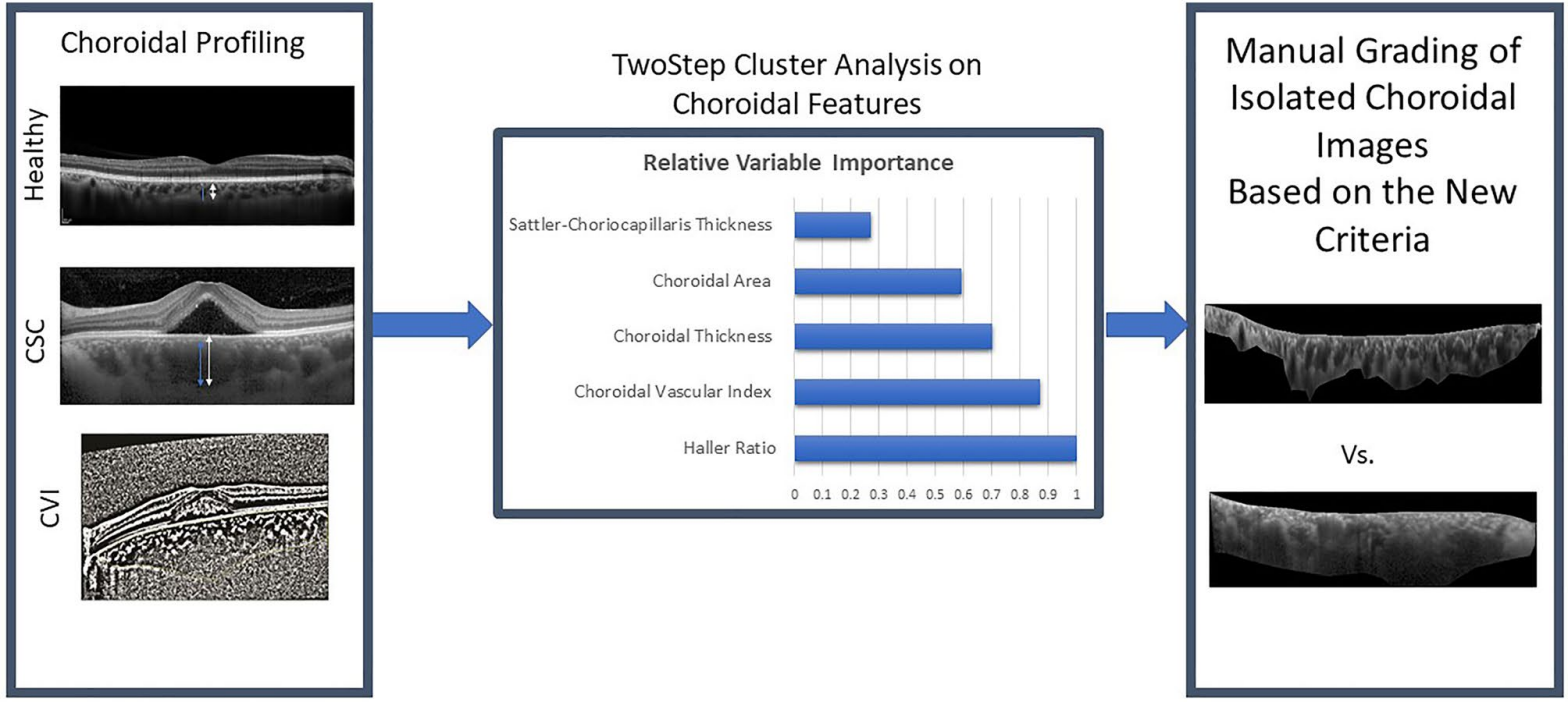
Schematic overview of the study design. Different choroidal features such as choroidal thickness, the thickness of haller ratio and choroidal vascular index (CVI) in central serous chorioretinopathy (CSC) patients and healthy subjects were extracted from OCT images and used in TwoStep cluster analysis. Afterwards, based on the relative variable importance (RVI) index of the cluster analysis, new clinical criteria were developed for diagnosis of pachychoroid. Two retina specialists were asked to label the isolated choroidal images based on these new criteria.

Images from a cohort of patients with a diagnosis of CSC or PPE were included in this cross-sectional and longitudinal observational study. The original study was approved by the Ethical Committee of the Iran University of Medical Sciences (IR.IUMS.REC.1400.429) and adhered to the tenets of the declaration of Helsinki. Informed consent was obtained from all participants. A schematic overview of the study design is shown in Fig. 1.

The diagnosis of CSC was established based on fundus examination and multimodal imaging, including fluorescein angiography and spectral domain OCT (SD-OCT).

An indocyanine green angiography was performed in suspected cases. Patients with PPE were selected from fellow eyes of patients with unilateral CSC and had to have retinal pigment epithelial (RPE) alterations in SD-OCT. Eyes with previous history of photodynamic therapy and patients with other retinal and choroidal diseases, including macular neovascularization (MNV), PCV, diabetic retinopathy, uveitis, choroidal tumors, and vitreoretinal interface disorders, were excluded. A group of sex- and age-matched healthy subjects from a previously published dataset^9^ were considered the controls. Subjects with refractive error beyond the range of −6 to + 5 diopters (D), media opacity precluding good quality imaging, and healthy controls with any abnormalities in retinal layers based on OCT were excluded from the study.

### Image acquisition

All subjects underwent EDI-OCT imaging of the macula using an SD-OCT device (Spectralis HRA-OCT; software version 5.2.0.3; Heidelberg Engineering, Heidelberg, Germany). In order to avoid diurnal variations, all imaging examinations were performed from 3 PM to 6 PM. A 6 mm by 6 mm and 8 mm by 8 mm foveal centered series of images were obtained, and horizontal b-scans centered on the fovea were exported for image processing. Low-quality images in which the choroid–scleral interface (SCI) was not discernible were excluded from the study.

### Measurement of choroidal indices

**Figure 2:**
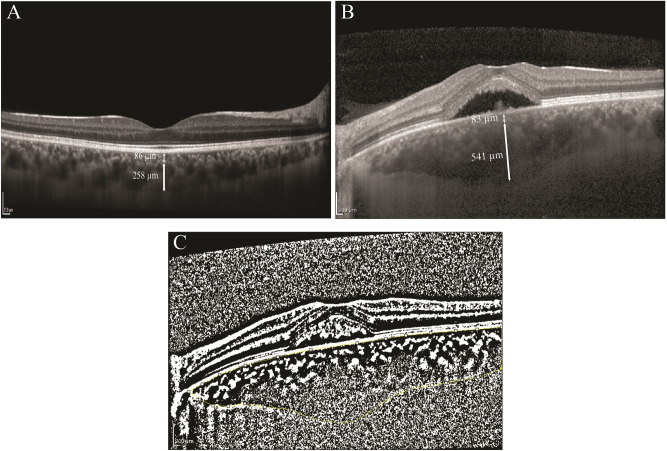
Choroidal thickness and vascularity index measurements. Haller layer (solid arrow) and the Sattler-choriocapillaris (dotted arrow) thickness manual measurements in enhanced depth imaging-optical coherence tomography of a healthy subject (**A**) and a patient with central serous chorioretinopathy (**B**). The Niblack’s autolocal thresholding method was used for binarization of the image. Then, subfoveal choroidal area was selected using the polygon tool (yellow dots). The dark pixels were selected using the color threshold tool and the area occupied by the dark pixels was defined as the luminal area. (**C**).

The EDI-OCT b-scan images were imported in Imagej software (http://imagej.nih.gov/ij/; provided in the public domain by the National Institutes of Health, Bethesda, MD, USA) for manual measurements. The choroidal thickness (CT) was measured perpendicularly from the external border of RPE/Bruch’s membrane complex to the inner border of SCI at the thickest part of the choroid in the subfoveal area (from 1500 µm nasal to 1500 µm temporal to the fovea). The thickness of the large choroidal vessels, known as the Haller layer, was determined at the thickest point of the subfoveal area from the inner border of the large choroidal vessels (measuring ≥ 100 µm) to the SCI as previously described^10^. The Haller ratio (HR) was defined by dividing the Haller thickness (HT) by the CT. The Sattler-choriocapillaris thickness (SCT) was determined by subtracting the Haller thickness from the total choroidal thickness at the same point (Fig. 2A,B). The subfoveal choroidal area (CA) was measured after outlining the choroid in the foveal and parafoveal areas from 1500 µm nasal to 1500 µm temporal to the fovea.

In order to assess the vascularity of the choroid, the choroidal vascularity index (CVI) was measured using a previously described method^4^. In brief, Niblack’s autolocal thresholding was used for the binarization of the image. Afterwards, subfoveal CA was selected using the polygon tool. The dark pixels were selected using the color threshold tool after converting the image to red, green, blue (RGB) image (Fig. 2C). The area occupied by the dark pixels was defined as the luminal area (LA). The CVI was calculated as follows:

$$\hspace{0.17em}\mathrm{CVI}=\hspace{0.17em}\frac{ LA}{CA}$$

All choroidal thickness measurements (i.e. choroidal thickness and Haller thickness measurements were performed by a skilled grader (RM) and rechecked by another retinal specialist (PA). In case of any dispute, the borders were outlined by consensus.

### Manual labeling of isolated choroid as pachychoroid or healthy choroid

**Figure 3:**
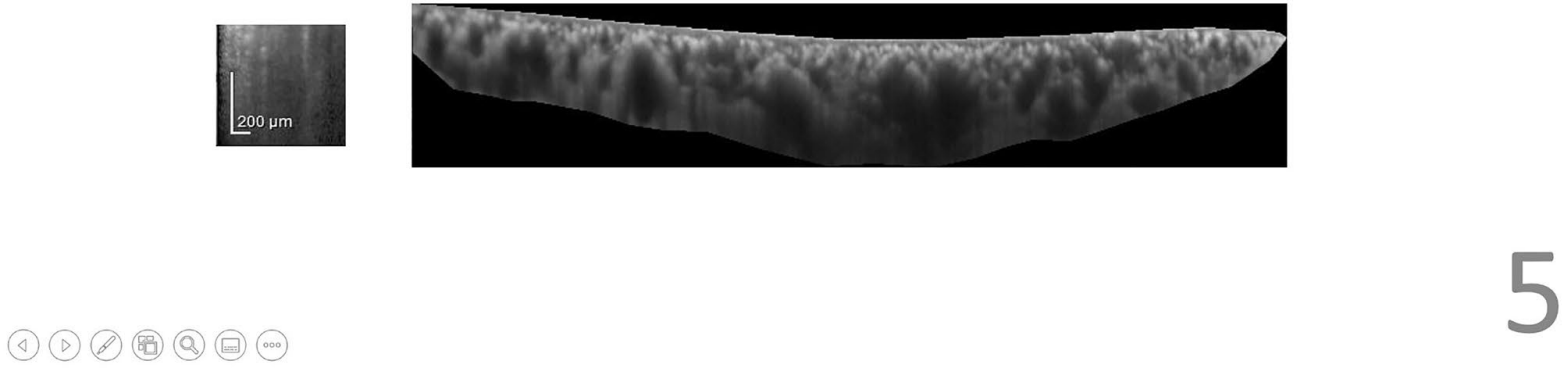
An example of isolated choroidal segmentation that was exported into a powerpoint slide for manual grading.

The segmented area of the choroid (without the overlying retinal layers) of 148 eyes from healthy subjects and CSC or PPE patients were randomly arranged in separate slides (Fig. 3) and manually graded as pachychoroid or healthy choroid by two independent skilled graders (PA and KGF). These eyes were considered as the “training dataset”, and the cluster analysis was performed on the variables extracted from these eyes. This grading on the training dataset was based on previously described criteria for pachychoroid^2^. A separate dataset consisting of 58 eyes from sex- and age-matched healthy subjects and CSC patients was used as the testing dataset. After cluster analysis, the grading of the “testing” dataset was performed based on the clinical criteria derived from the results. Manual grading was performed by observation of the images, and no additional quantitation tools, including calipers, were used.

### Statistical analysis

All statistical analyses were performed using SPSS (version 22.0, IBM Corp., Armonk, NY, USA) and Excel (2013, Microsoft Inc., Redmond, WA, USA). Continuous data, presented as mean ± standard deviation (SD) and percentage (%), are used for the presentation of categorical data. Cohen's kappa was used to determine the inter-rate reliability. The demographics and choroidal features were compared between the two groups using a two-tailed Student t test and Chi-square test. An adjusted P-value < 0.05 was considered statistically significant.

### Cluster analysis

Segmentation or cluster analysis is an exploratory method of analysis that aims to identify hidden structures within data. It finds homogenous clusters of cases based on distribution of input variables provided. Cluster analysis can be done even if the actual grouping of the cases is not known. More specifically, TwoStep cluster analysis recognizes the grouping by combining the two of the best available approaches: first pre-clustering then the hierarchical method. The latent associations between patients with complex set of characteristics can be revealed by this analysis tool^6^.

TwoStep cluster analysis with different combinations of variables and a pre-fixed number of clusters (cluster 1 & 2) was performed using SPSS software. The sensitivity and specificity of each method were determined by comparing the cluster membership variable (output of cluster analysis) with the original labeling of patients and healthy subjects. TwoStep cluster analysis provides a relative variable importance (RVI) for each variable in differentiating between clusters. From all statistically available imaging variables, two clinically feasible variables (choroidal thickness and Haller ratio) were selected for the cluster analysis step of developing the clinical criteria. Then, ROC curve analysis was performed to determine the optimum cut-off point of the correct cluster membership variable.

To evaluate the accuracy of the developed clinical criteria, a testing dataset of 29 eyes of 23 patients with CSC or PPE and 29 healthy subjects was used. The testing dataset cases were separate from those included in the training set.

## Results

The training dataset included 74 eyes from 74 healthy subjects and 74 eyes of 59 patients with CSC or PPE. Table 1 summarizes the demographic findings and the details of choroidal quantifications in the training dataset. Five eyes (6.8%) had PPE, and 69 eyes (93.2%) had CSC. In 66 eyes (89.2%) of the CSC and PPE group and all healthy eyes, the maximum choroidal thickness was in the subfoveal point.

**Table 1:** Demographics and choroidal metrics of patients with central serous chorioretinopathy or pachychoroid pigment epitheliopathy and healthy subjects in the training dataset.

Variable	Healthy	CSC or PPE	P-value
Age: mean ± SD (range) years	42.38 ± 8.97 (30.00–61.00)	41.05 ± 6.84 (30.00–68.00)	0.314*
Sex (%male)	64.9%	75.7%	0.150 ^†^
Choroidal area: mean ± SD (range) µm^2^	850,982.04 ± 197,896.04 (374,607.84–1,363,112.75)	1,411,928.99 ± 348,232.06 (848,774.51–2,393,774.51)	< 0.001*
Maximum choroidal thickness: mean ± SD (range) µm	286.57 ± 75.78 (118.00–455.00)	521.82 ± 120.70 (331.00–826.00)	< 0.001*
Haller thickness: mean ± SD (range) µm	198.01 ± 59.00 (79.00–340.00)	460.09 ± 116.70 (267.00–761.00)	< 0.001*
Haller ratio	0.70 ± 0.07 (0.42–0.85)	0.88 ± 0.05(0.73–0.97)	< 0.001*
Sattler and choriocapillaris thickness: mean ± SD (range) µm	93.96 ± 29.57 (39.00–163.00)	61.73 ± 25.45 (18.00–156.00)	< 0.001*
Choroidal vascular index: mean ± SD (range)	71.86 ± 2.40 (66.01–77.76)	64.44 ± 3.28 (57.85–77.78)	< 0.001*

*CSC* central serous chorioretinopathy, *PPE* pachychoroid pigment epitheliopathy, *SD* standard deviation.

*Student t test.

^†^Chi-square test.

**Figure 4:**
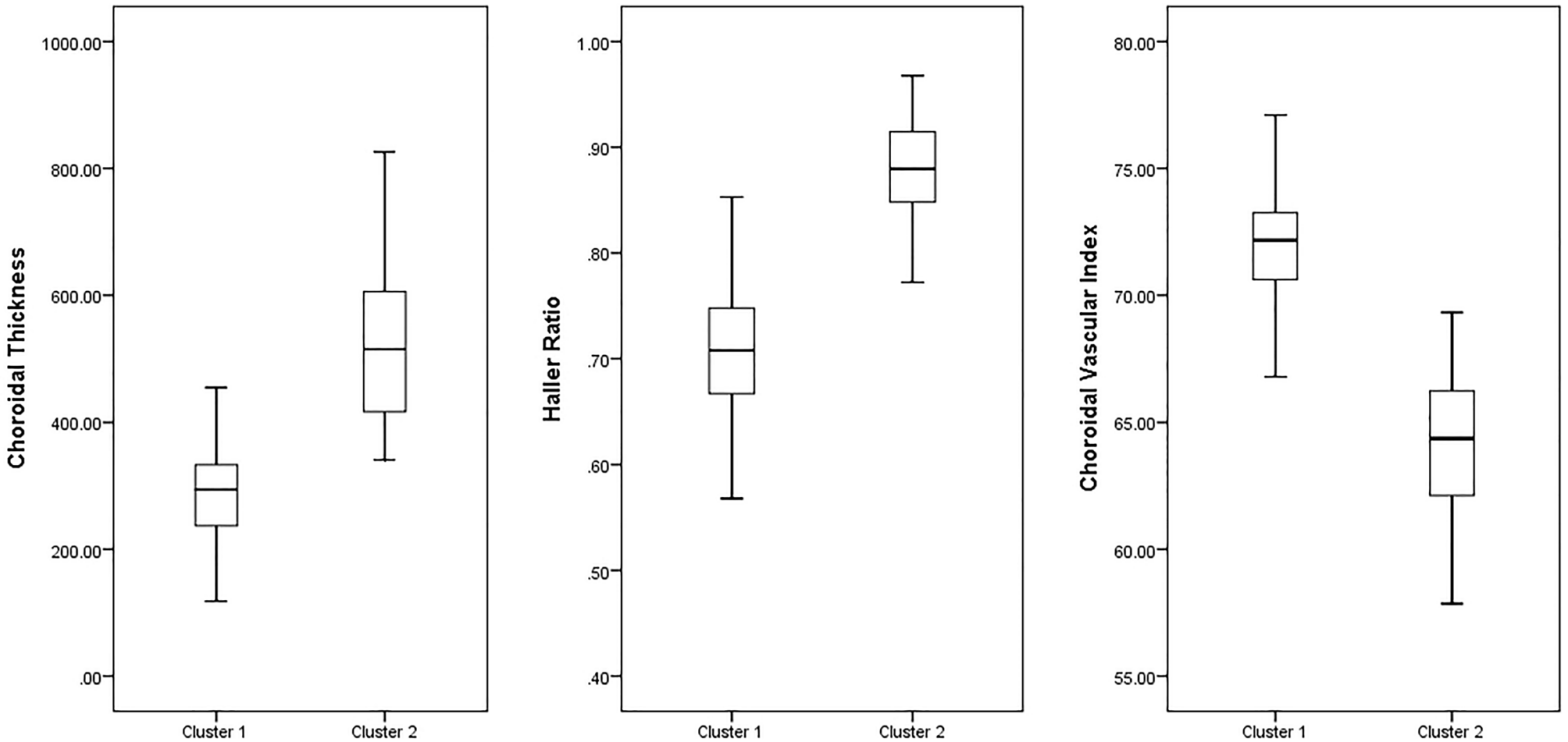
The box and whisker plot of the difference in input variables of TwoStep cluster analysis between cluster 1 and 2.

**Figure 5:**
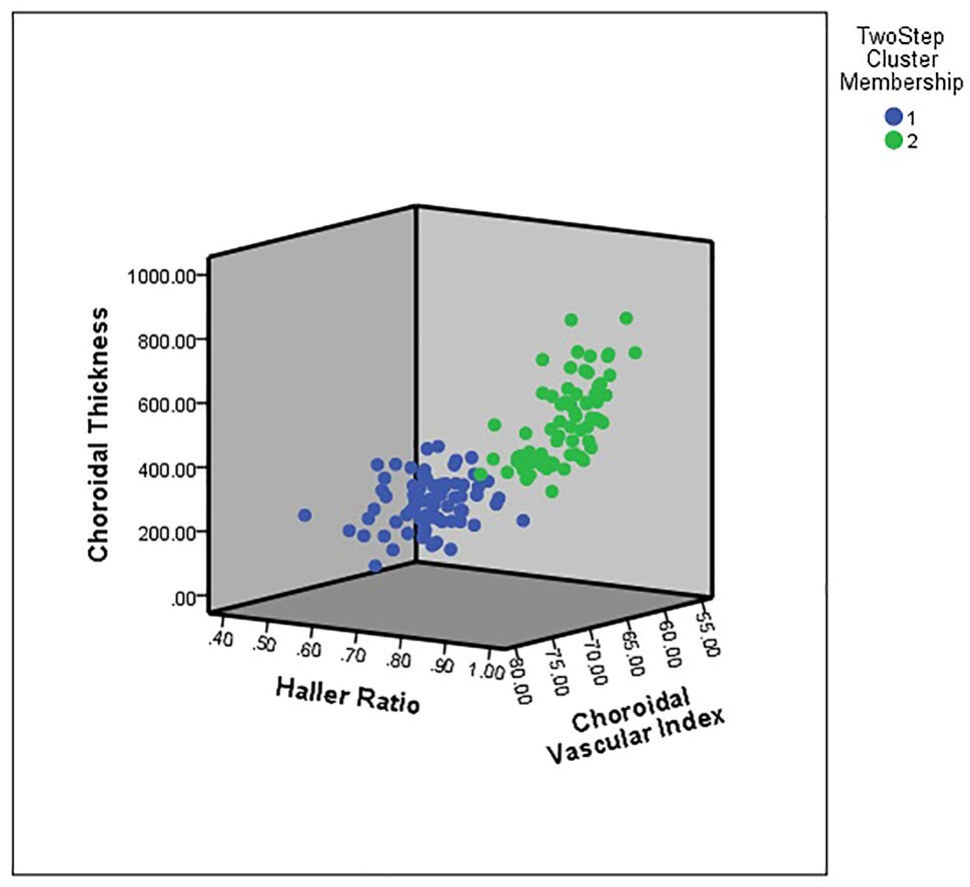
The t-SNE scatter plot based on three variables showing the successful process of clustering.

TwoStep cluster analysis with different combinations of the indices was performed, and the best result for the identification of pachychoroid was obtained for a combination of CT, HR, and CVI, with a correct classification rate of 0.993 (95-CI: 0.980–1.000) (Table 2, Figs. 4, 5). Based on RVI, the most important differentiating factors between the two clusters were HR (RVI: 1.0), CVI (RVI: 0.87), CT (RVI: 0.70), CA (RVI: 0.59), and SCT (RVI: 0.27), respectively. The accuracy of the manual graders (KGF and PA) to correctly classify the training dataset (isolated choroid only) based on previously reported criteria was 0.762 (95-CI: 0.693–0.831) (Table 3). The Cohen's kappa of inter-rater reliability was 0.502 for the training dataset.

**Table 2:** Accuracy, sensitivity, and specificity of the choroidal measurements in various combinations.

Combination of variables	Accuracy	Sensitivity	Specificity
CA, CT, HR, HT, SCT and CVI	0.980 (95-CI: 0.957–0.1)	1.000 (95-CI: 0.938–1.000)	0.959 (95-CI: 0.882–0.990)
CA, CT, HR, SCT and CVI	0.980 (95-CI: 0.957–1.000)	0.986 (95-CI: 0.918–1.000)	0.973 (95-CI: 0.900–0.998)
CA, CT, HR and CVI	0.986 (95-CI: 0.968–1.000)	0.986 (95-CI: 0.918–1.000)	0.986 (95-CI: 0.919–1.000)
CT, HR and CVI	0.993 (95-CI: 0.980–1.000)	1.000 (95-CI: 0.938–1.000)	0.986 (95-CI: 0.919–1.000)
CT and HR	0.959 (95-CI: 0.927–0.991)	0.945 (95-CI: 0.862–0.982)	0.973 (95-CI: 0.900–0.998)

*CA* choroidal area, *CI* confidence interval, *CT* choroidal thickness, *CVI* choroidal vascular index, *HR* Haller ratio, *HT* Haller layer thickness, *SCT* Sattler-choriocapillaris thickness.

**Table 3:** Manual grading on the training dataset.

Manual grader	Accuracy	Sensitivity	Specificity
Grader 1	0.762 (95-CI: 0.693–0.831)	0.689 (95-CI: 0.576–0.783)	0.836 (95-CI: 0.732–0.904)
Grader 2	0.762 (95-CI: 0.693–0.831)	0.892 (95-CI: 0.798–0.946)	0.630 (95-CI: 0.515–0.732)

*CI* confidence interval.

**Figure 6:**
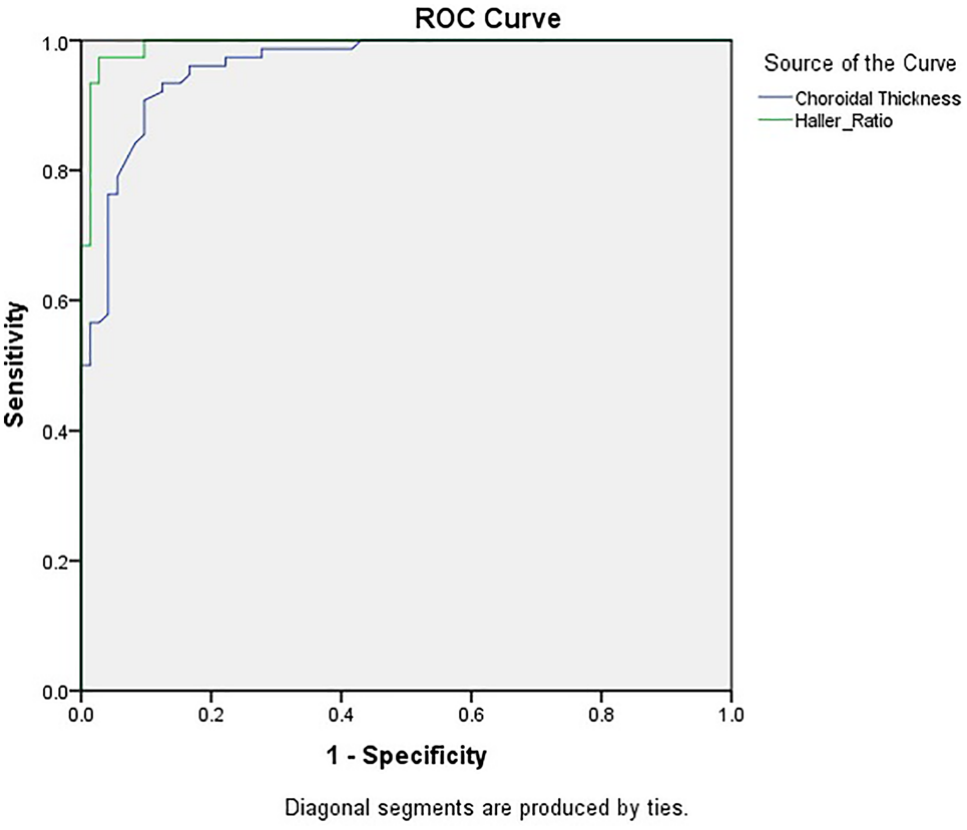
The receiver operating characteristic curve of the choroidal thickness and Haller ratio for the cluster membership variables obtained from cluster analysis.

Based on ROC curve analysis on the cluster membership variable of TwoStep cluster analysis, the areas under the curve (AUCs) for the Haller ratio and choroidal thickness were 0.993 and 0.960, respectively (Fig. 6). A cutoff point of 389 µm for CT and 0.79 for HR was found as the best clinical criteria for differentiation of healthy and pachychoroid cases.

Table 4 summarizes the demographic findings and the details of choroidal quantifications in the testing dataset.

**Table 4:** Demographics and choroidal metrics for patients with central serous chorioretinopathy or pachychoroid pigment epitheliopathy and healthy subjects in the testing dataset.

Variable	Healthy	CSC or PPE	P-value
Age: mean ± SD (range) years	45.24 ± 10.11 (30.00–60.00)	44.34 ± 9.70 (28.00–65.00)	0.732*
Sex (%male)	79.3%	86.2%	0.487^†^
Maximum choroidal thickness: mean ± SD (range) µm	319.86 ± 79.09 (177.00–549.00)	477.79 ± 86.63 (334.00–666.00)	< 0.001*
Haller ratio	0.70 ± 0.06 (0.48–0.79)	0.86 ± 0.03(0.79–0.94)	< 0.001*

*CSC* central serous chorioretinopathy, *PPE* pachychoroid pigment epitheliopathy, *SD* standard deviation.

*Student t test.

^†^Chi-square test.

The clinical criteria achieved an accuracy of 0.895 (95-CI: 0.815–0.974) with a sensitivity of 0.793 (95-CI: 0.611–0.904) and specificity of 1.000 (95-CI: 0.854–1.000). The accuracy of the manual graders (KGF and PA) to correctly classifying the testing dataset (isolated choroid only) based on the new clinical criteria was 0.789 (95-CI: 0.684–0.895) and 0.807 (95-CI: 0.705–0.909), respectively (Table 5). The Cohen's kappa of the inter-rater reliability was 0.895 in the testing dataset.

**Table 5:** Manual grading on the testing dataset.

Manual Grader	Accuracy	Sensitivity	Specificity
Grader 1	0.789 (95-CI: 0.684–0.895)	0.793 (95-CI: 0.611–0.904)	0.786 (95-CI: 0.600–0.900)
Grader 2	0.807 (95-CI: 0.705–0.909)	0.793 (95-CI: 0.611–0.904)	0.821 (95-CI: 0.638–0.924)

*CI* confidence interval.

## Discussion

In this study, a new unsupervised machine learning approach was used to differentiate choroidal features in patients with CSC or PPE from healthy subjects based on EDI-OCT. The exploratory statistical method of cluster analysis helped to rank the differentiating importance of various choroidal indices.

Previous studies on artificial intelligence applications in this field are mainly focused on image classification and diagnosing CSC based OCT^11^, OCT angiography^12^, and fundus photography^13^. Unsupervised machine learning using cluster analysis has been used extensively in medical literature to develop differentiative features of various diseases^14–16^. For example, cluster analysis has been used to find different clinical faces of sleep apnea^17^. In ophthalmology, however, its use has been limited to small studies in uveitic diseases and macular degeneration^8,18^. In a recent study in Chinese population, cluster analysis was used to develop diagnostic criteria for Fuchs’ uveitis syndrome with high sensitivity and specificity^7^. Cluster analysis has also been used to distinguish pachychoroid neovasculopathy from age-related macular degeneration^18^. To the best of our knowledge, our study is the first to use the exploratory technique of cluster analysis to find the most important components of choroidal profiles differentiating pachy- from healthy choroids.

The term “pachychoroid” is used extensively in the literature to describe a common structural and functional finding in several pathologic conditions referred to as “pachychoroid spectrum”. Although the term literally means “thickened choroid”, a clear definition for pachychoroid has yet to be determined. In a comprehensive literature review, Spaide^19^ reported that there was no stated definition for pachychoroid in the majority of studied articles. Interestingly, when the disease was defined, the criteria were often equivocal. In an attempt to provide a consensus on the definition of pachychoroid, Castro-Navarro et al.^2^ concluded that the presence of large vessels under an area of reduced or absent choriocapillaris and not thickened choroid per se is the most prominent feature of pachychoroid. However, they did not provide a clear definition for vascular dilation. In this study, patients with CSC or fellow eyes with SD-OCT confirmed pigment epitheliopathy were included to ensure that all eyes have pachychoroid^20^. The results showed that a subfoveal CT of 389 µm is the best cutoff for differentiating pachychoroid from healthy eyes. This is similar to the cutoff reported by Lehmann et al., which observed the best threshold measure for differentiating CSC from healthy choroid at 395 µm^21^. Although a combination of different choroidal metrics yielded the highest accuracy, the findings of the present study revealed that HR was the most important single factor for categorizing a patient as pachy- or healthy choroid. Previous studies have shown that the Haller thickness is increased in eyes with CSC^22,23^. However, similar to pachychoroid, there is no clear cutoff for differentiating increased Haller thickness in CSC and other pachychoroid diseases^19^. In addition, it has been shown that the choriocapillaris and Sattler layer thickness is decreased in pachychoroid disease^2^. Therefore, we proposed a new index, the Haller ratio, to more appropriately reflect the relation of choroidal larger vessels to the total choroidal thickness and found a ratio of 0.79 as the best single factor differentiating eyes with CSC or PPE from healthy eyes.

Several quantitative measurements have been proposed for differentiating pachychoroid disease from healthy eyes^2^. However, in a busy clinical practice, the application of these measurements is not easy. Based on cluster analysis, two clinically feasible parameters, CT and HR, were extracted. A choroidal thickness cutoff of 389 µm can be easily determined based on the OCT printout scale (e. g. 2 × 200-µm scale in Spectralis OCT printout). Similarly, an HR of 0.79 is a fast and practical measurement in an OCT printout (80% of the choroidal thickness). Our proposed clinical criteria achieved a higher performance in the classification of the isolated choroidal features and showed more reliable consistency with higher inter-rater reliability compared to previously published criteria^2^.

This study has some limitations. We included eyes with CSC or PPE, which are major categories of pachychoroid disease; however, other pachychoroid diseases may have different choroidal metrics. In addition, we did not analyze the angiographic, fundus photo and en face OCT findings. Moreover, we did not use automatic segmentation methods for delineation of choroid that might affect our results. The relatively small number of participants can be another source of bias especially regarding the included cases in training and testing datasets.

In conclusion, the results of this study show that a combination of CT, HR, and CVI is the most important factor for differentiating pachy- from healthy choroids. In addition to features previously described in the literature (e.g., choroidal thickness and CVI), the Haller ratio is another important differentiating factor in patients with pachychoroid in CSC or PPE. The definition of pachychoroid is not clear in the literature. Therefore, the findings of the current study can be helpful in developing clinical criteria to diagnosis pachychoroid based on choroidal features alone in patients without obvious signs of CSC. Further studies based on machine learning approaches with a larger sample size and more ethnic diversity in the subjects might help develop a universal definition for the pachychoroid spectrum.
